# A multimodal assessment of balance in elderly and young adults

**DOI:** 10.18632/oncotarget.7758

**Published:** 2016-02-26

**Authors:** Gregory W. King, Eduardo L. Abreu, An-Lin Cheng, Keyna K. Chertoff, Leticia Brotto, Patricia J. Kelly, Marco Brotto

**Affiliations:** ^1^ Human Balance and Ambulation Research Laboratory, School of Computing and Engineering, University of Missouri, Kansas City, MO, USA; ^2^ Muscle Biology Research Group (MUBIG), School of Nursing and Health Studies, University of Missouri, Kansas City, MO, USA; ^3^ Current address: Bone-Muscle Collaborative Sciences, College of Nursing and Health Innovation, University of Texas, Arlington, TX, USA

**Keywords:** aging, balance, strength, troponin, posture, Gerotarget

## Abstract

Falling is a significant health issue among elderly adults. Given the multifactorial nature of falls, effective balance and fall risk assessment must take into account factors from multiple sources. Here we investigate the relationship between fall risk and a diverse set of biochemical and biomechanical variables including: skeletal muscle-specific troponin T (sTnT), maximal strength measures derived from isometric grip and leg extension tasks, and postural sway captured from a force platform during a quiet stance task. These measures were performed in eight young and eleven elderly adults, along with estimates of fall risk derived from the Tinetti Balance Assessment. We observed age-related effects in all measurements, including a trend toward increased sTnT levels, increased postural sway, reduced upper and lower extremity strength, and reduced balance scores. We observed a negative correlation between balance scores and sTnT levels, suggesting its use as a biomarker for fall risk. We observed a significant positive correlation between balance scores and strength measures, adding support to the notion that muscle strength plays a significant role in postural control. We observed a significant negative correlation between balance scores and postural sway, suggesting that fall risk is associated with more loosely controlled center of mass regulation.

## INTRODUCTION

Falling poses a significant health risk for the elderly. As the baby boom generation moves into the over age 65 demographic, the number of age-related falls will increase, highlighting the need for accurate and effective fall risk assessment tools. Unlike other age-related health problems that are often organ- or system-specific, fall risk is influenced by a complex range of factors involving neurological, cardiovascular, musculoskeletal, somatosensory, and vestibular components [1-4]. Risk is further exacerbated by external risk factors such as poor lighting, trip hazards, and uneven or slippery surfaces [2]. Fall risk assessment based on any of these factors individually can be misleading and lead to less effective intervention strategies. Accurate fall risk assessment should include evaluation of risk factors from multiple sources [5]. Researchers in selected studies and settings have used multifactorial fall risk interventions [5-7]; however, the relationship among fall risk variables is complex and still not well understood, and more work is necessary for a better understanding that will lead to more efficient fall prevention interventions.

Quiet standing is a balance-related activity that involves contributions from both the musculoskeletal and proprioceptive systems, and represents an effective model in which to study their combined influence on balance regulation and fall risk. More specifically, we selected a quiet standing task for the present study because the postural sway outputs from the proprioceptive system are likely related to upper and lower extremity muscle strength and to serum biomarkers for muscle health, skeletal muscle-specific troponin T, sTnT.

Skeletal muscle troponin, a complex formed by three proteins (Troponin C, Troponin I, and Troponin T), is a critical component to muscle contraction, as it provides a binding site for calcium ions (Ca^2+^) and subsequent actin-myosin crossbridge formation. Age-related changes in connective tissue surrounding the muscle can impair muscle integrity, resulting in leaking of troponins into the bloodstream and reduced muscle contraction efficacy. Therefore, the presence of skeletal muscle Troponin T (sTnT) in the blood is a potentially valuable biomarker for muscle health and sarcopenia, which is an important risk factor for falls [8-10]. In fact, previous work has shown that serum sTnT levels are sensitive to inactivity and exercise as demonstrated by decreased levels of sTnT among older adults after a 10-week strength-training regimen [9].

Age-related changes in upper and lower extremity strength are expected, given these age-related muscular effects. Previous work has shown clear evidence of age-related declines in leg extension strength, defined as maximal leg extension torque, which relate to falls in older adults [11]. Grip force, an indicator of upper extremity strength is a potential but weaker predictor for fall risk because grip does not directly employ the muscles used in balance and mobility [11].

Postural sway, or horizontal movement from the center of gravity, is another component of balance impairment, as it reflects the postural control system's ability to stabilize the moving center of mass. A large body of work has demonstrated age effects on sway, including age-related increases in sway amplitude [12-16]. While there are central factors that contribute to age-related changes in sway, peripheral effects such as sarcopenia, coupled with larger proportions of Type I and the loss of the more powerful Type II muscle fibers in older adults, is also important in the development of age-related sway patterns [17].

Fall risk is difficult to measure among older adults as it requires retrospective or prospective monitoring of falls for long periods of time. To avoid this limitation, many studies implement estimates of fall risk using qualitative instruments. For example, the Tinetti Balance Assessment (TBA) is an instrument used to estimate fall risk with a balance score categorizing participants as high, moderate, or low fall risk. TBA balance scores were used in this work as a proxy for true fall risk.

To identify the potential for an interactive fall risk assessment strategy, we investigated the relationship among sTnT, muscle strength, sway, and balance score in a group of healthy, community-dwelling elderly adults when compared to a group of healthy young adults. We hypothesized that elderly adults, compared to young, would exhibit altered indicators of fall risk: (1) larger levels of sTnT and sway, reduced levels of upper and lower extremity strength, and lower balance scores; and (2) that changes in STnT, sway, and strength would have a significant correlation with balance scores.

## RESULTS

### Age effects on sTnT, sway, strength, and balance scores

**Figure 1:**
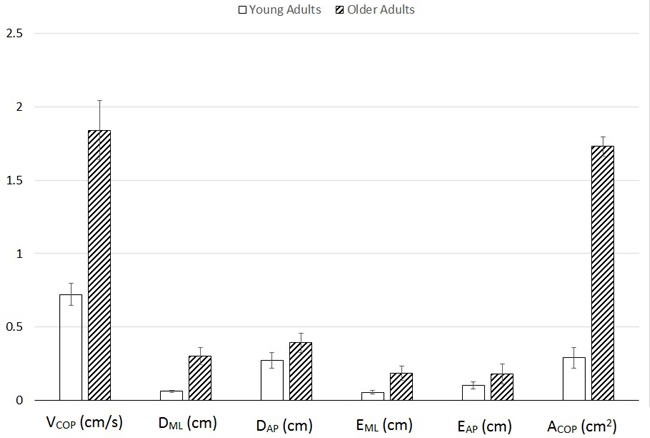
Means and standard errors of sway variables, including center of pressure path velocity (V_**COP**_), average medial-lateral (D_**ML**_) and anterior-posterior (D_**AP**_) center of pressure displacement, root-mean-squared error between center of pressure and center of mass for medial-lateral (E_**ML**_) and anterior-posterior (E_AP_) directions, and center of mass area (A_**COP**_).

**Figure 2:**
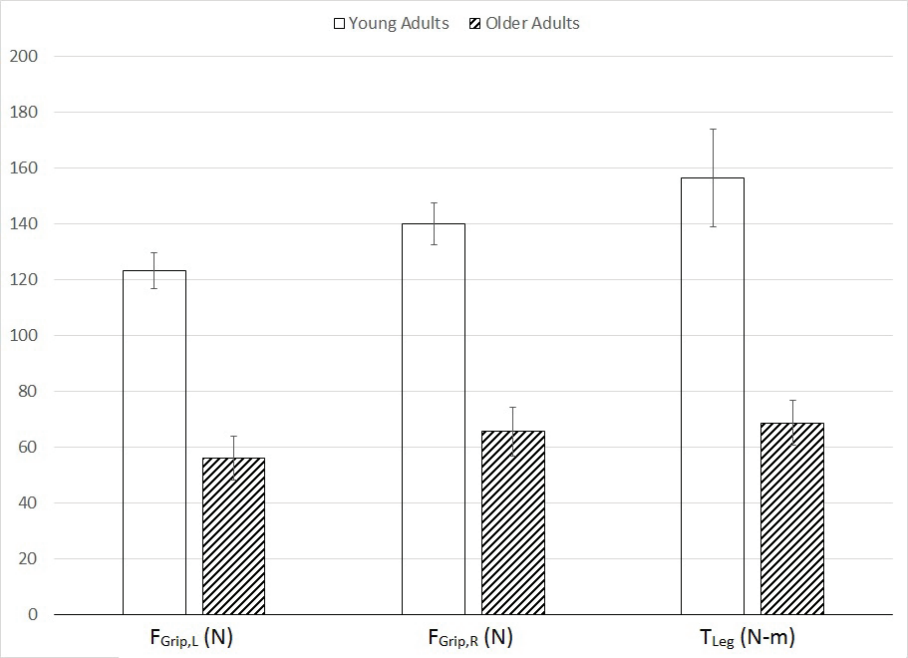
Means and standard errors of strength variables, including maximal left (F_**Grip,L**_) and right (F_**Grip,R**_) grip forces and maximal leg extension torque (T_**Leg**_).

**Figure 3:**
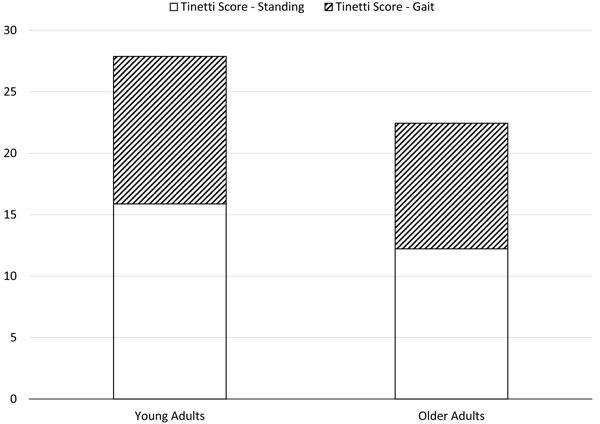
Tinetti balance scores in young and older participants. Note that the sum of standing (T_Stand_) and gait (T_Gait)_ scores is equal to the overall balance score (T_Total_).

Mann-Whitney *U* tests revealed age-related increases in postural sway variables including center of pressure (COP) average velocity [V_COP_: *U* = 0.00, *p* < .001], mean medial-lateral COP displacement [D_ML_: *U* = 4.00, *p* < .001], and sway area [ACOP: *U* = 9.00, *p* < .05] (Figure 1). Age-related decreases in strength were observed in both left hand [F_Grip,L_: *U* = 0.00, *p* < .001] and right hand [F_Grip,R_: *U* = 0.00, *p* < .001] grip force, as well as maximal leg extension torque [T_Leg_: *U* = 3.00, *p* < .001] (Figure 2). Additionally, age-related decreases in all three balance scores were observed, including standing score [T_Stand_: *U* = 0.5, *p* < .001], gait score [T_Gait_: *U* = 8.0, *p* < .01], and total score [T_Total­_: *U* = 0.00, *p* < .001] (Figure 3). Although there was a 7% age-related increase in sTnT, this result did not reach statistical significance [*U* = 32.0, *p* = .743].

**Table 1:** Mean (SD) of all study variables in young adult (YA) and older adult (OA) participants

	YA	OA
T_Stand_	15.880 (0.354)	12.220 (3.383)^a^
*T_Gait_	12.000 (0.000)	10.220 (1.856)^b^
T_Total_	27.880 (0.354)	22.440 (4.187)^a^
*sTnT (pg/ml)	40.088 (18.965)	42.889 (26.728)
F_Grip,L_(N)	123.154 (18.017)	56.060 (23.615)^a^
F_Grip,R_(N)	140.056 (21.412)	65.514 (26.357)^a^
T_Leg_ (N-m)	156.563 (49.520)	68.589 (24.067)^a^
V_COP_ (cm/s)	0.722 (0.213)	1.841 (0.607)^a^
D_ML_ (cm)	0.062 (0.018)	0.301 (0.176)^a^
D_AP_ (cm)	0.272 (0.149)	0.392 (0.192)
E_ML_ (cm)	0.056 (0.036)	0.184 (0.147)
E_AP_ (cm)	0.105 (0.069)	0.181 (0.206)
A_COP_ (cm^2^)	0.290 (0.196)	1.73 (0.196)^c^

Significant age effects are denoted by superscripts in the OA column

a *p* < .001

b *p* < .01

c *p* < .05

sTnT was negatively correlated with T_Gait_ (rho = −0.590, p = .094) among older adult participants.

### Relationship between balance scores and sTnT, sway, and strength measures

Correlation analyses by age group revealed that sTnT is negatively correlated with T_Gait_ [rho = −0.590] in the older group; however this result did not reach statistical significance *p* = .094).

Significant positive correlations were observed between balance scores and all measures of strength (Table 2). Significant negative correlations were observed between balance scores and several measures of postural sway, including V_COP_, D_ML_, D_AP_, and A_COP_ (Table 3).

**Table 2:** Spearman correlation coefficients between balance scores and strength measures

	F_Grip,L_	F_Grip,R_	T_Leg_
T_Stand_	0.836^a^	0.846^a^	0.883^a^
T_Gait_	0.731^a^	0.810^a^	0.605^b^
T_Total_	0.858^a^	0.883^a^	0.806^a^

a *p* < .01

b *p* < .05

**Table 3:** Spearman correlation coefficients between TBA scores and measures of postural sway

	V_COP_	D_ML_	D_AP_	A_COP_	E_ML_	E_AP_
T_Stand_	−0.778^a^	−0.798^a^	−0.310	−0.645^a^	−0.400	−0.113
T_Gait_	−0.686^a^	−0.696^a^	−0.502^b^	−0.806^a^	−0.382	−0.341
T_Total_	−0.809^a^	−0.815^a^	−0.417	−0.744^a^	−0.425	−0.210

a *p* < .01

b *p* < .05

## DISCUSSION

Each year millions of adults over age 65 experience a fall [18]. Unfortunately, the problem does not end with the fall itself, since falls have now become the leading cause of both fatal and nonfatal injuries among elderly adults in the United States [19]. In 2013 alone, 2.5 million nonfatal falls among elderly adults were treated in emergency departments and over 730,000 of these patients were hospitalized [18], generating direct medical costs of $34 billion dollars [20].

While the etiology of falls is multifactorial, research on risk tends to be reductionist. To our knowledge, to date, no others studies have simultaneously looked into several potential contributors or risk factors for falls ranging from strength to balance to a newly proposed serum biomarker. To address this gap, we conducted the simultaneous measurements of sTnT, left and right hand grip strength, leg extension strength, and postural sway to evaluate their relationship to fall risk as assessed using standing balance (T_Stand_), gait (T_Gait_), and total (T_Total_) scores from the Tinetti Balance Assessment.

We observed significant age-related changes in nearly all variables studied, largely supporting our first hypothesis. Elderly adults exhibited right grip strength, left grip strength, and leg extension strength levels that were 53.2%, 54.5%, and 56.2% lower, respectively, than those in young adults. This result clearly demonstrates age-related loss of muscle function (sarcopenia) that is well documented in the aging literature [21, 22], despite the lack of treatment and prevention, and even awareness.

All balance scores (T_Stand_, T_Gait_, T_Total_) were significantly lower among older adults in comparison to young. The older adults in our study had an average overall balance score (T_Total_) of 22.4 out of 28, which corresponds to the “Moderate” fall risk category. Young adults scored, on average, 27.9 out of 28, a nearly perfect score that corresponds to the “Low” fall risk category. While this clearly indicates an age-related increase in fall risk among our participants, care must be taken when interpreting this result: nearly all young adults received perfect scores, resulting in very small variation in comparison to older adults that may have contributed to a detection bias of the *U* tests and correlation analyses used.

We observed an age-related increase in several of the postural sway variables measured, including center of pressure path velocity (V_COP_), average medial-lateral center of pressure displacement (D_ML_), and sway area (A_COP_). The findings for V_COP_, D_ML_, and A_COP_ collectively represent an age-related increase in center of pressure magnitude, which has been observed elsewhere [23, 24] and may be interpreted as an age-related change characterized by less precise control of the moving center of mass [25-27]. Although not statistically significant, we observed a trend suggesting an age-related increase in E_ML_, which represents the difference between medial-lateral center of mass and center of pressure movements. In essence, older adults use a postural control scheme that is characterized by larger oscillations of the center of pressure around the center of mass (Figure 5).

Of the direction-specific center of pressure parameters (mean displacement and RMS error), only the medial-lateral ones exhibited an age-related increase. This result suggests that, given the lack of age effects in the anterior-posterior directional COP parameters, the differences in sway velocity and area are attributed mainly to age-related changes in medial-lateral components of sway. This finding carries implications for lateral stability among older adults, particularly given the emphasis in the literature on laterally-directed stability and falls [28-30].

Interestingly, we did not observe any significant age effects on sTnT, although there was a trend towards an age-related increase. One explanation for this observation relates to possible differences in activity levels between young and older adults. While we did not measure activity levels, anecdotal evidence from speaking with participants suggests that elderly adults in our study were active. If the young adults (who were college-aged) were relatively sedentary, this may have minimized the differences in sTnT between age groups. Future studies should include information about participants' activity levels in order to control for their potential confounding effect. Furthermore, while we apply a strict statistical comparison to detect age-related changes in sTnT, one might also extrapolate from the cardiac troponin literature that a 7% increase in serum levels would in fact be considered pathological. Given that we are defining and proposing a new biomarker, and until several studies are conducted and a more detailed behavior for sTnT is determined, the possibility remains that a 7% difference could be of high physiologic significance.

We observed significant correlations between balance scores and measures of strength, sway, and sTnT that largely support our second hypothesis. Although not reaching statistical significance, we found a negative correlation between gait balance score (T_Gait_) and sTnT, suggesting that increased serum levels of troponin are associated with a decline in balance performance (at least on the gait portion of the TBA). Further analysis revealed that this effect was only present among older adults, as they were the only subjects who scored less than perfect on the gait portion of the TBA. This result has exciting implications for using sTnT as a biomarker for fall risk in older adults. However, care should be taken in interpreting this result: the variability in sTnT contributed to overlap between age groups, meaning there were some young adults with higher levels of sTnT who still received perfect scores on the gait portion of the TBA. This clearly suggests that age-related processes apart from sarcopenia play a role in older adults' increased fall risk. Although we did not measure these in the current study, age-related declines in proprioception and vision may have also contributed to older adults' lower gait scores. The variation in sTnT also demonstrates the need for future studies to have larger sample sizes.

**Figure 4:**
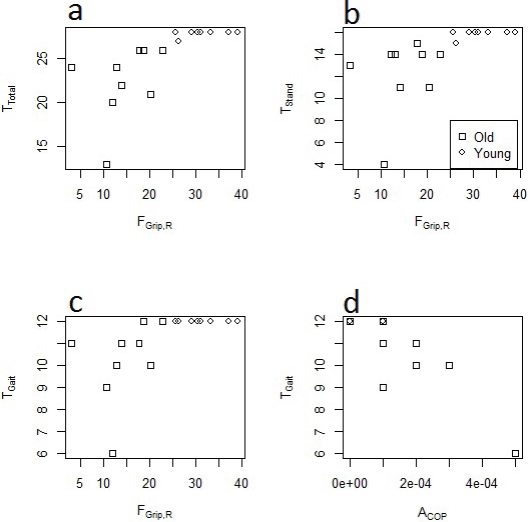
Scatter plots depicting relationships between right grip force (F_Grip,R_, in lb). a. overall balance score (T_Total_), b. standing balance score (T_Stand_), and c. gait score (T_Gait_); and between d. center of pressure area (A_COP_) and T_Gait_.

All of our strength measures were positively correlated with balance scores, clearly demonstrating the link between muscle strength and balance ability. Although we did not perform regression analyses within age groups, visual analysis of balance scores plotted as a function of grip strength (Figure 4) demonstrate clear separation between the age groups and suggest that the significant relationship between grip strength and balance score can be attributed to variability among older adults. Additionally, these plots suggest the presence of a threshold level of strength below which there is an impact on balance ability. This is consistent with the idea of strength reserve in that a difference exists between maximal strength and task-specific strength requirements. Therefore, age-related decreases in maximal strength did not have an impact on task performance (balance ability, in this case) until maximal strength approaches or falls below task requirements [31].

All postural sway variables were negatively correlated with balance scores, with sway velocity, average displacement, and sway area reaching statistical significance. Graphical observation (Figure 4d) suggests that sway area in particular was a good predictor of balance ability, a result that is consistent with the age-related [23, 24] and fall-related [32, 33] increases in sway reported elsewhere. As suggested above, this result may again relate to more loosely-controlled adjustments to the moving center of mass [25-27]. Graphical observation of the relationship between sway area and T_Gait_ score (Figure 4) also suggests that most of the variation in these variables is attributed to elderly adults. This effect is similar to that observed between sTnT and T_Gait_ in that the young adults participating all received perfect T_Gait_ scores; however there was much less variation in sway area in comparison to sTnT, to the extent that nearly all participants with sway area greater than ∼100 mm^2^ had less than perfect gait balance scores.

Despite its unique nature, our study has some limitations. Given its preliminary nature, our work is limited by small sample size and by the fact that measurements were only performed during a single visit to the laboratory. Further work with larger sample sizes is required to better understand the results observed here and to determine their effects when monitored over longer periods. The preliminary nature of this work also limited us to studying general age effects on the outcome variables. Given the goal of extending this work to develop a fall risk assessment paradigm, future studies should involve large enough sample sizes of elderly participants to provide an adequate comparison between those with a history of falls and age-matched controls.

An additional limitation stems from the use of the Tinetti Balance Assessment as the sole fall risk estimation tool. While the TBA has shown to be highly reliable and sensitive to fallers [34], true fall risk assessment requires prospective or retrospective tracking of participants to monitor actual fall incidence, which was not done in the current study.

In contrast to these limitations, the uniqueness of our study arises from our observation that age-related effects in all of the measurements studied, including a trend toward increased sTnT levels, increased indices of postural sway, reduced upper and lower extremity strength, and reduced balance scores. Furthermore, we observed trends indicating a negative correlation between Tinetti scores, which assess fall risk, and sTnT levels, suggesting the use of skeletal muscle troponin T as a potential biomarker for fall risk among older adults. This finding supports our previous hypothesis that sTnT could function as a robust muscle biomarker because of its strategic location in the sarcomere, and its presence at high levels in the blood can easily be interpreted as muscle wasting or muscle damage. A significant positive correlation was observed between balance scores and strength measures, adding support to the notion that muscle strength plays a significant role in postural control. Finally, we observed a significant negative correlation between balance scores and postural sway, suggesting that fall risk is associated with more loosely controlled regulation of body center of mass. Our studies, while preliminary in nature, start to direct us that the multifactorial nature of falls might be revealed through a combination of strength, balance, and biochemical analyses. We propose now to further advance these studies by applying them to larger cohorts of younger and older adults and with different forms of interventions ranging from resistance training to nutritional ones.

**Figure 5:**
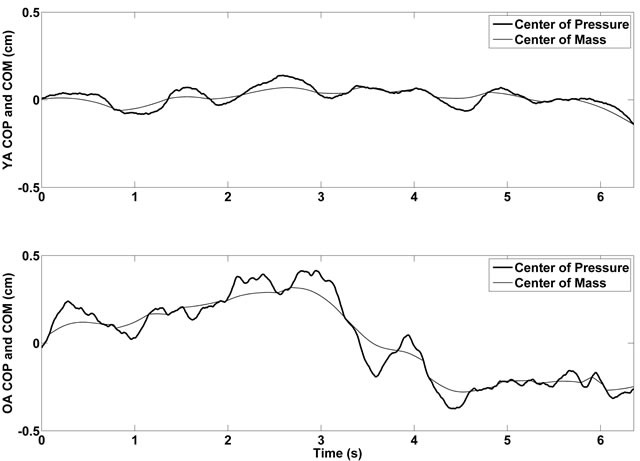
Representative medial-lateral center of pressure and center of mass time histories for young (top panel) and older (bottom panel) participants.

## MATERIALS AND METHODS

### Subjects

Eleven elderly adults, all female, (M = 88.3, SD = 4.6 years) and eight young adults, all female, (M = 22.4, SD = 3.1 years) participated in the current study. Elderly adults were recruited from community sites at which the researchers had previously worked, and four had a previous history of fall. Young adults were recruited from the University of Missouri-Kansas City (UMKC) student body. All participants denied having any significant musculoskeletal, neurological, or cardiovascular conditions that would impair their ability to comply with study tasks, defined as the ability to stand or walk without assistance for 30 seconds at a time. UMKC's Institutional Review Board approved all study procedures.

### Procedures and measurements

Each participant completed a single visit to UMKC's Human Motion Laboratory and, after signing informed consent, participated in the following procedures: (1) phlebotomy; (2) upper extremity grip strength test; (3) lower extremity knee strength test; (4) balance assessment.

To determine serum levels of sTnT, a phlebotomist drew blood from participants into serum separator tubes. The blood was allowed to clot for two hours at room temperature and centrifuged at 1000 x g before serum samples were collected and stored at −80°C until sTnT levels were determined using a commercially available ELISA kit (USCN Life Science Inc., Houston, TX, USA) following the manufacturer's instructions.

To evaluate upper body strength, grip force (F_Grip,R_, F_Grip,L_) was assessed by asking each participant to perform three maximal grip contractions with their right and left hands, with three minutes of rest between contractions, using a grip strength dynamometer (model DHS-176, Detecto Scale Co., MO, USA). For each hand, grip strength was determined as the highest force value of the three trials.

To measure lower extremity strength, leg extension torque (T_Leg_) was assessed by asking each participant to perform maximal isometric leg extensions with the dominant leg while positioned on a multi-mode dynamometer (Biodex Medical Systems, Inc., Shirley, NY, USA). Participants performed three contractions of three seconds duration each; the maximum measured torque among the contractions was recorded as T_Leg_.

Balance was assessed with the Tinetti Balance Assessment (TBA), a clinical tool used to measure balance impairment based on performance in several activities including sitting, standing, turning around, and gait [35]. With the exception of gait, participants completed all components of the TBA with their feet in contact with a six-axis force platform (AMTI, Watertown, MA, USA). Two independent observers scored all TBA components; scores were aggregated into standing balance (T_Stand_), gait (T_Gait_), and total (T_Total_) for each participant. Force plate data was captured at a sampling rate of 1000 Hz, and a separate center of pressure (COP) profile was recorded for each component of the standing balance portion of the TBA. While all components of the TBA were included in the aggregate fall risk score, only the eyes-open, quiet stance portion of the TBA was included in the quantitative sway analysis as stationary conditions are required to extract the biomechanical parameters of interest.

### Data analysis

All force platform data was processed with MATLAB V14 (Mathworks, Natick, MA, USA). We extracted several different measures of postural sway from COP time histories, including average sway velocity (V_COP_), medial-lateral (D_ML_) and anterior-posterior (D_AP_) average sway displacement, and COP area (A_COP_). Additionally, we generated transverse plane center of mass (COM) time histories for each trial using a zero-point-to-zero-point integration algorithm [36], which were subsequently used to calculate root-mean-squared error (RMSE) between medial-lateral (E_ML_) and anterior-posterior (E_AP_) COM and COP time histories.

To test the first hypothesis that age-related changes would be observed in sway, sTnT, strength, and TBA score, Mann-Whitney *U* tests were performed on all outcome variables (upper and lower extremity strength measures, skeletal muscle troponin T levels, postural sway measures, and Tinetti scores) with age group as the independent variable.

To test the second hypothesis that a correlation existed between TBA score and measures of strength, sTnT, and sway, Spearman correlations were conducted to explore potential relationships among the variables. Spearman correlations were also conducted to examine this relationship by age group. All statistical analyses were conducted using the statistical software SAS version 9.3, with significance set at *p* < 0.05.
